# The Time Is Up: Compression of Visual Time Interval Estimations of Bimodal Aperiodic Patterns

**DOI:** 10.3389/fnint.2017.00017

**Published:** 2017-08-08

**Authors:** Fabiola Duarte, Luis Lemus

**Affiliations:** Primate Neurobiology Laboratory, Instituto de Fisiología Celular, Neurociencia Cognitiva, Universidad Nacional Autónoma de México Ciudad de México, Mexico

**Keywords:** time interval, aperiodic, visual compression, acoustic dominance, psychophysics, audiovisual, bimodal

## Abstract

The ability to estimate time intervals subserves many of our behaviors and perceptual experiences. However, it is not clear how aperiodic (AP) stimuli affect our perception of time intervals across sensory modalities. To address this question, we evaluated the human capacity to discriminate between two acoustic (A), visual (V) or audiovisual (AV) time intervals of trains of scattered pulses. We first measured the periodicity of those stimuli and then sought for correlations with the accuracy and reaction times (RTs) of the subjects. We found that, for all time intervals tested in our experiment, the visual system consistently perceived AP stimuli as being shorter than the periodic (P) ones. In contrast, such a compression phenomenon was not apparent during auditory trials. Our conclusions are: first, the subjects exposed to P stimuli are more likely to measure their durations accurately. Second, perceptual time compression occurs for AP visual stimuli. Lastly, AV discriminations are determined by A dominance rather than by AV enhancement.

## Introduction

The ability to estimate the duration of an event is fundamental for many of our sensory and motor behaviors such as talking, walking or even cooking, and dancing. However, such ability can be altered by magnitude, beat, sensory congruity, or saliency of an event (Xuan et al., 2007; van Wassenhove et al., 2008; Grube and Griffiths, 2009; Kösem et al., 2012; Kanai et al., 2017). Nevertheless, to understand how the brain estimates the duration of processes, linked to human behavior, such as how music intervals create the perception of the beat (Pashler, 2001) and rhythm (Geiser et al., 2014), requires studying temporal estimations of aperiodic (AP) patterns in different contexts and models. For instance, whether AP stimuli other than musical rhythms affect time interval perception, is still open for debate.

Empirical evidence suggests that the representation of time intervals is far from having a single neural machinery. Some have suggested that different internal clocks can create relative, rather than absolute temporal estimations (Buhusi and Meck, 2009). Others have proposed figural coding for specific complex stimuli (Povel and Essens, 1985), or hybrid mechanisms (Merchant et al., 2008). More recent models lean towards the existence of diverse and independent sensory and motor networks (Karmarkar and Buonomano, 2007; Merchant et al., 2013; Hardy and Buonomano, 2016) that perhaps function based on the sampling rate of each sensory modality (Welch and Warren, 1980). For instance, the auditory system is more appropriate at perceiving temporal modulations (i.e., within the range of flutter: 5–50 Hz; Bendor and Wang, 2007; Lemus et al., 2009a,b), and the most suitable for estimating time intervals (Goldstone and Goldfarb, 1964; Wearden et al., 1998; Repp and Penel, 2002; Burr et al., 2009). In contrast, visual fusion occurs above ~16 Hz. Such reduced sampling rate sometimes produces perceptual time intervals as either compressed (Grondin and McAuley, 2009), or prolonged (Schiffman and Bobko, 1974; Kanai et al., 2006).

Evidence suggests that time interval perception is hierarchically built across the sensory pathways, eventually converging in higher cortical areas (Stauffer et al., 2012; Rammsayer et al., 2015). Multimodal interactions in association cortices achieve the perception of time intervals (Grahn et al., 2011; Kuroda et al., 2013), but only after different sensory inputs are recalibrated into a common signal (Heron et al., 2009). However, many brain areas, such as the motor and premotor cortices, the pre-supplementary and supplementary motor cortices, the basal ganglia and the cerebellum, are involved in time interval perception (Sakai et al., 1999; Jäncke et al., 2000; Lewis and Miall, 2003). Furthermore, Merchant et al. (2013) have shown that, during a synchronization-continuation task in monkeys, single unit recordings of neurons from the medial premotor cortex show tuning activity to time intervals in the range of milliseconds. Neurons mostly exhibit sustained activity for stimuli of periodic (P) patterns and different time intervals but not for AP patterns (i.e., random inter-pulse-intervals; Merchant et al., 2011). Moreover, only a few studies have reported how AP metrics alter the human time interval perception (Povel and Essens, 1985; Hébert and Cuddy, 2002). Likewise, experiments in monkeys trained to discriminate two consecutive tactile frequencies demonstrated that, based on a firing rate code, neurons of the second somatosensory cortex keep track of the metrics of AP stimuli (Salinas et al., 2000). Based on that finding, it is now that we understand how cortical neurons code AP information based on firing rate rather than on inter-spike time intervals as was demonstrated for the discrimination of AP intervals in other cortical areas (Luna et al., 2005; Rossi-Pool et al., 2016). Alternatively to the time interval coding based on firing rates modulations, it has been observed that the duration of complex rhythmical sequences is estimated in short term memory circuits of association cortices that recruit different categorical pre-wired time interval representations (Bengtsson et al., 2009). In spite of all this evidence, it is still not clear how the duration of AP intervals are discriminated by different sensory modalities.

In the present psychophysical study, we tested the human timing perception of P and AP structures during visual (V), acoustic (A) and audiovisual (AV) interval discriminations. We hypothesized that the perceived duration of a stimulus is affected when it is presented in an AP sequence. We found that time intervals of AP structures were perceived as compressed during V discriminations, whereas A, and AV accuracies and reaction times (RTs) were globally affected. Moreover, we found no evidence of AV integration. To our knowledge, these results are the first to show how the metrics of AP stimuli correlate with their perceived durations across sensory modalities.

## Materials and Methods

### Participants and Ethics Statement

Thirty-one students from the UNAM (naïve to the experiment; mean age and standard deviation = 23.6 ± 4.3; 15 women) gave written consent to participate in a two-alternative-forced-choice (2AFC) psychophysical task. All the participants were right-handed and declared no physical impairment, i.e., normal or corrected-to-normal vision and no audition deficits. The experiments were carried out under the guidelines of The Code of Ethics of the World Medical Association and supervised and approved by the Bioethical Committee of the Institute of Cellular Physiology, UNAM.

### Setup

The experiments took place inside a psychophysics booth. Each participant sat in a chair ~5 cm behind a table, facing a laptop (Windows 7 Professional, Dell Precision M6800 Intel^®^ Core™ i7-4710MQ, 2.5 GHz, RAM 16 GB, 64-bit OS; 17.3″ screen 1920 × 1080; 60 Hz refreshing rate). The V stimuli consisted of trains of pulses stored in AVI clips at a rate of 60 frames per second and presented at the center of the screen at 60 cm V distance. Each V pulse consisted of a 50 ms 4°radius gray circle. To avoid flicker fusion during V stimulation, we restricted the likelihood of appearance of two consecutive pulses to no more than an instant frequency of 15 Hz. The A stimuli consisted of WAV files containing trains of pulses. Each pulse was a 50 ms 1 kHz tone at ~65 dB SPL. Stimuli were delivered binaurally through active noise-blocking headphones (Bose^®^ QC25), along with a constant white-noise background at ~55 dB SPL. Finally, AV stimuli were created by superimposing WAV sounds to their dynamically equivalent AVI clips (WAV were shifted +90 ms to create perceptual simultaneity). Participants used their right hand to submit a response through the laptop’s keyboard. The task was programmed in LabView (SP1 130 64-bits, National Instruments^®^).

### Behavioral Task

**Figure 1:**
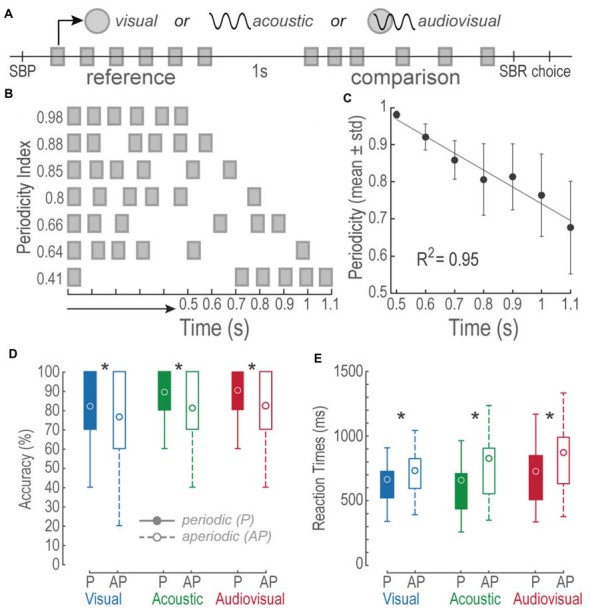
Bimodal interval discrimination task. **(A)** Events during visual (V), acoustic (A) and audiovisual (AV) trials. A single trial initiated by pressing and holding down the spacebar (SBP). Then, a 0.8 s-long reference stimulus consisting of a train of six V, A, or AV pulses distributed periodically, was followed by an interstimulus of 1 s. Later, a comparison stimulus (see “Materials and Methods” Section for details on the comparison structures and durations) was presented and subjects had to release the spacebar (SBR) and report whether it was longer or shorter than the reference by pressing the upward or downward arrow key (choice), respectively. Squares: 50 ms V, A or AV pulses described by the upper symbols.** (B)** Depictions of comparison stimuli patterns and their interval durations. Notice that the shorter the time intervals, the higher their periodicity index. **(C)** Linear regression of the mean periodicity indexes as a function of time intervals. Error bars = SD. **(D)** Boxplots of accuracies of 31 subjects for each of the modalities and tested conditions. Circles represent the mean accuracies. Filled boxes represent periodic conditions. Open boxes represent the aperiodic condition. P, periodic; AP, aperiodic. Asterisks denote differences between P and AP intramodal values. **(E)** Same as in **(D)** but for reaction times (RTs).

The task consisted on discriminating interleaved V, A and AV trials of two consecutive stimuli. Figure 1A describes the sequence of events during the task. Briefly, in a single run a 0.8 s regular V, A, or AV reference stimulus was delivered after the subjects initiated the trial by pressing the spacebar (SBP); subjects held the spacebar down with their right index finger during the rest of the trial. After a 1 s interstimulus period, a P or AP comparison stimulus of the same modality, but 85% of the trials of a different duration (42% higher duration), was presented. Finally, as soon as the comparison period ended, the participants expressed their decision by releasing the spacebar (SBR) and pressing one of two buttons with their right index finger (Choice). They pressed the upward arrow button when they perceived the comparison stimulus to be longer than the reference and the downward arrow button otherwise. The RT was measured from the end of the comparison stimulus to the SBR. If the participants released the spacebar before the end of the stimulus the trial was aborted. All of the 31 subjects participated in all the different experimental conditions; each one performed a total of 420 trials (140 trials per modality condition). For every modality, 70 P and 70 AP trials resulted from repeating each possible time interval 10 times (see below).

### Design of Periodic and Aperiodic Patterns

We created V, A and AV stimuli consisting of trains of six-pulses presented in P or AP sequences. Stimuli durations ranged from 0.5 s to 1.1 s on increments of 0.1 s. The first and last pulses delimited the interval duration of a stimulus; however, in an AP stimulus, the four intermediate pulses were randomly distributed. In contrast, P pulses were evenly spaced throughout the entire stimulus. To characterize the temporal structure of a stimulus, we determined its periodicity index by dividing the geometric mean of its inter-pulse-intervals by the geometric mean of the P stimulus of the same duration. Thus, the periodicity index was:

(1) $$PI=\frac{Geometric\;mean[AP]}{Geometric\;mean[P]}$$

the geometric mean being:

(2) $$GM=\sqrt[{n}]{x1\cdot x2\cdot \ldots xn}$$

where *x*1 corresponds to the first inter-pulse interval and *n* to the total number of inter-pulse-intervals. Figure 1B depicts some examples of stimuli with their respective periodicity index. Figure 1C shows a monotonic increment of AP variability as a function of time interval increments (Linear fit *R*^2^ = 0.94, permutation test: *p* < 0.001).

### Data Analysis

Accuracy and RT distributions of P and AP experiments were compared by paired *t*-test analyses (alpha = 0.05). The averages for both accuracies and RTs excluded uncertainty trials where comparison stimuli lasted 0.8 s.

**Figure 2:**
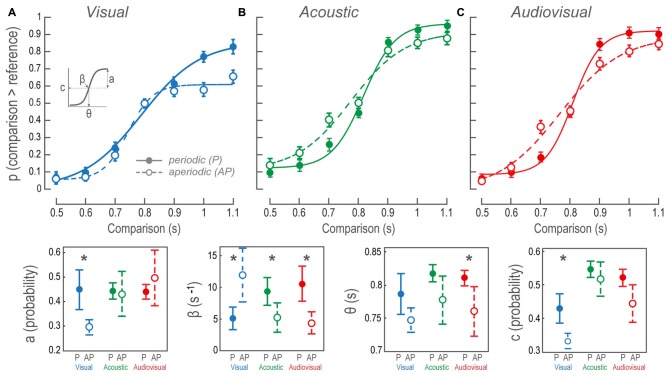
Psychophysical performance during the bimodal interval discrimination task. **(A–C)** Sigmoidal fit to the probability of perceiving comparisons as being longer than the reference, as a function of comparison durations. Data points and error bars show participants’ overall mean and standard error of the mean, respectively. Each data point of the AP condition shows the performance averaged over 10 different stimuli patterns, in contrast to the P conditions where only one structure was possible. Continuous lines and closed circles: discrimination of P stimuli. Dashed lines and open circles: discrimination of AP stimuli. **(Lower panels)** The contrast between mean and confidence intervals for the sigmoidal fit parameters (a, β, θ and c, as inset in **(A)**; see “Materials and Methods” Section). P, periodic; AP, aperiodic. Asterisks denote intramodal differences.

To detect behavioral biases during the time interval discriminations of AP stimuli, we performed psychometric analyses on the performance by fitting a hyperbolic tangent (tanh) to the probability of perceiving a comparison stimulus as longer than the reference, at each time interval. The tanh function delivers a good description about the psychophysical performance through four parameters expressed as follows:

(3) P(S2>S1)=a⋅tanh[β(s2−θ)]+c

where S2 and S1 are the durations of the comparison and reference stimuli. The parameter *β* is the first derivative at the inflection point which indicates the steepness of the transition between the categories longer and shorter than the reference. The amplitude parameter *a* describes the minimum and maximum performance (also the distance from chance). The parameter *θ* or X0 is the *x*-value at the inflection point where time intervals bias into longer or shorter than the reference categories. Additionally, *c* is Y0 or *y*-value at the inflection point that reveals performance biases towards one or another category. For all sigmoidal fits in our experiment, *χ*^2^, *Q* > 0.05. Figure 2 (lower panels) depicts the confidence intervals of the sigmodal’s parameters used to evaluate differences between P and AP conditions. Non-overlapping confidence intervals proved differences within a parameter; however, overlapping intervals did not disregard differences, and thus we performed an error reduction method to find statistical significance:

(4) $$(\bar{X_{1}}-\bar{X_{2}})-1.96\cdot \sqrt{SE_{1}^{2}+SE_{2}^{2}}>0$$

Finally, to characterize the relationship between behavior and the magnitude of the stimuli’s aperiodicity, we performed a Pearson’s Correlation analysis between accuracy and periodicity indexes at each time interval. This analysis was also conducted for P and AP normalized RTs of each participant at every time interval. Significant values were obtained shuffling data 1000 times and recalculating the correlation index at each run (permutation test, *p* < 0.01). All of the analyses were coded in Matlab (The Mathworks Inc., Natick, MA, USA).

## Results

During a two-time-interval discrimination task, we evaluated whether human subjects perceived V, A and AV AP stimuli as lasting longer or shorter than P stimuli. Figure 1D shows boxplots of the overall accuracies during each experiment, expressed in percentage. We conducted paired *t*-tests to compare intra-modal accuracies between P and AP conditions. All comparisons proved differences: V P vs. V AP, *t*_(185)_ = 3.39, *p* < 0.01; A P vs. A AP, *t*_(185)_ = 6.18, *p* < 0.01; AV P vs. AV AP, *t*_(185)_ = 5.82, *p* < 0.01. These differences demonstrated a higher accuracy in P trials.

Figure 1E shows boxplots of RTs expressed in milliseconds. We conducted paired *t*-tests to intra-modally compare the RTs between P and AP conditions. Once again, all comparisons revealed differences: V P vs. V AP, *t*_(185)_ = −3.79, *p* < 0.01; A P vs. A AP, *t*_(185)_ = −4.99, *p* < 0.01; AV P vs. AV AP, *t*_(185)_ = −7.09, *p* < 0.01. During AP trials RTs were slower as compared to P trials. These results suggest that non-periodic stimuli affected accuracies and RTs during V, A and AV discriminations.

To better understand the observed differences in accuracies, we contrasted the regression parameters of P and AP psychometric curves. Afterward, at each time interval, we analyzed whether the aperiodicity magnitudes affected accuracies and RTs. These results are described next.

### Vision Compressed the Perceived Duration of Aperiodic Intervals

Figure 2A illustrates sigmoidal fits of the performance as a function of V P and AP comparison time intervals. The fit to V AP data revealed a bias towards reporting the comparisons as shorter than the reference at all time intervals. The contrast of the V P and V AP sigmoidal fits revealed that the regression parameters *a*, *β* and *c* of the *tanh* function were different (Figure 2, lower panels; see “Materials and Methods” Section and inset in Figure 2A for descriptions on the statistics of current and subsequent results). The AP *β* was larger than the P, indicating that during V AP discriminations the transition from perceived shorter to longer time intervals occurred faster, steepening the psychometric curve. In this case, categories longer and shorter than the reference both fell quickly into a performance plateau in which the subsequent time intervals were confined. In other words, all time intervals within such categories presented a similar perceptual probability. In contrast, the transition between categories in V P was smoother because performance gradually increased at each time interval. Furthermore, consistent with the overall V AP performance, AP *a*, was smaller than P *a*. The reason for this was that near threshold (during the comparison of 0.9 s) performance reached a plateau at a probability of ~0.6, as we had previously mentioned. It is important to acknowledge that accuracies for comparisons lower than the reference were higher than those of V P. These two observations are consistent with a perceptual compression of V AP time intervals because subjects consistently referred comparisons as lower than the reference. Additionally, AP *c* was biased downwards, so the inflection point at the ordinates indicated that, compared to V P, both categories were considered more frequently lower than the reference.

### The Acoustic Time Interval Estimations Were Less Accurate for Aperiodic Structures

Figure 2B shows the sigmoidal fits of the population performance during A P and A AP tests as a function of the comparison time intervals. The intersection among *β* confidence intervals (Figure 2, lower panels), show that AP *β* was statistically lower than P *β*, indicating that the transition from the category shorter to the larger than the reference was smoother than during P discriminations. Since neither *c* nor *a* parameters were different, then *β* differences meant that A AP comparisons were affected at all time intervals showing no bias towards any particular category. This can be interpreted as a larger decision threshold for A AP discriminations.

### Audiovisual Interval Discriminations Were Explained by Auditory Dominance

Figure 2C shows AV P and AV AP sigmoidal fits. We observed differences in *θ* and *β* values because AP discriminations were less accurate; particularly during time interval differences near threshold. The AP *θ* was lower because of a noticeable decline in performance over the comparison of 0.7 s, whereas the 0.9 s time interval was slightly better. Nonetheless, such bias could not be explained by a perceptual time interval compression since 0.7 s had a ~0.4 probability of being classified as higher than the reference, in contrast to the ~0.2 V AP likelihood during the same time interval. This particular case had a straightforward interpretation: near threshold, non-periodic intervals created a higher impact on performance. The same explanation stood for the decrease in the *β* parameter since the transition between categories was smoother during AV AP.

Furthermore, to test whether AV conditions were dominated by V or A perception, we compared cross-modally the accuracies for P and AP conditions. We only found differences between V P vs. AV P conditions; *t*_(185)_ = 5.48, *p* < 0.01; and V AP vs. AV AP; *t*_(185)_ = 3.12, *p* < 0.01.

Together AV P and AV AP discriminations resembled those of A, as opposed to V conditions, cross-modal analyses of their regression parameters did not exhibit differences. Moreover, given that AV performances were not different from A performances, but differed from V conditions, AV discriminations were better explained by auditory dominance. Furthermore, since the AV did not show any improvements in performance, no phenomenon of multisensory integration could be claimed.

### The Perceived Duration of a Stimulus Correlated to the Regularity of its Structure

**Figure 3:**
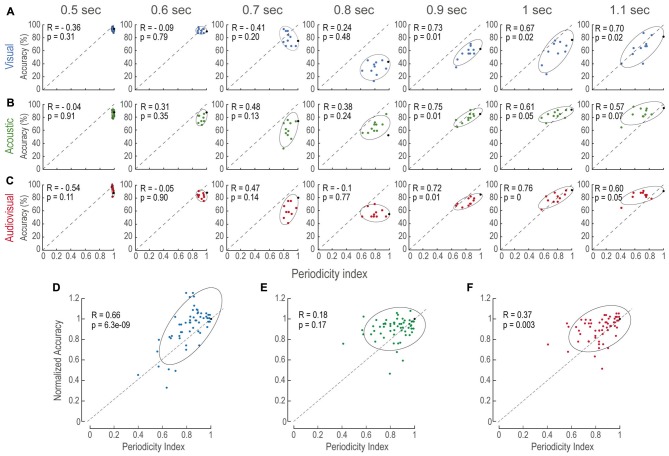
Accuracy increases as a function of periodicity. **(A–C)** Accuracy as a function of periodicity index for every modality (panels in horizontal) and time interval (panels in vertical). In all panels, each data point is the averaged accuracy of the population during the presentation of a single AP stimulus pattern. Black points depict accuracy of P stimuli (i.e., periodicity indexes of 1.0). Ellipses are 2σ-contour for a two-dimensional Gaussian fit to data distributions. Upper left corners show Pearson correlations (R) between accuracy and periodicity, and their corresponding *p*-values (permutation test *n* = 1000). **(D–F)** Normalized accuracy to the performance at each P interval to directly observe the effects of periodicity rather than interval duration. In all panels, dashed diagonals represent a linear fit with ordinate at 0 and correlation between accuracy and periodicity index of 1.0.

To further evaluate the relationship between accuracy and periodicity, we calculated the Pearson’s Correlation index (R) for every modality and time interval (Figures 3A–C). We found that most of the comparison intervals in the longer category (i.e., 0.9, 1 and 1.1 s) were correlated (*p* < 0.05). In contrast, no correlation existed at intervals where the comparisons were shorter than the reference (i.e., 0.5, 0.6 and 0.7). This result indicated that performance was biased by the degree of the regularity within each structure, as it could be witnessed at short time intervals that mostly consisted of nearly regular patterns, which will be addressed in the “Discussion” Section.

Similarly, to understand how modalities were affected by aperiodicity at each time interval we sought to reduce the effect of time interval differences with the reference stimuli. We reasoned that, since subjects discriminated P stimuli based on time intervals alone and not on structure, normalizing the performance on AP tests to the P of the same duration would reduce the effect of time differences (Figures 3D–F). We found that aperiodicity affected V and AV alone (linear regression, *p* < 0.01; 0.8 s intervals, where no decision criterion existed, were excluded). Figure 3E shows that normalized A accuracy was not globally correlated to aperiodicity. However, this was possibly due to the 1.1 s time interval that presented no correlation. These results suggested that the magnitude of the aperiodicity within V, A and AV structures impacted on their estimated duration.

### Effects of the Aperiodicity on the Reaction Times

**Figure 4:**
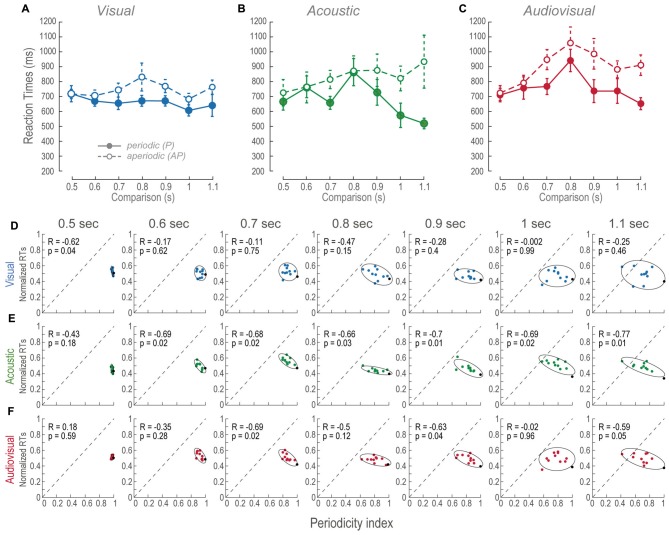
RTs across modalities and time intervals. **(A–C)** Mean RTs of the population as a function of the comparison durations during P and AP conditions. Error bars are the standard error of the mean. **(D–F)** Normalized mean RTs for P and AP patterns at each time interval. Ellipses are 2σ-contour for a two-dimensional Gaussian fit to data distributions. The upper left corners show Pearson correlations (R) between RTs and periodicity indexes, and their corresponding *p*-values (permutation test *n* = 1000). Closed circles and continuous lines: P stimuli. Open circles and dashed lines: AP stimuli. Gray dashed diagonals represent a linear fit with ordinate at 0 and correlation between RTs and periodicity index of 1.0.

As we had previously mentioned all RTs in AP conditions were different from the P. Such differences consisted in increased AP RTs (Figures 4A–C). In order to elucidate the effect of aperiodicity in the RTs, we performed a correlation between the means of the subjects’ normalized RTs and periodicity indexes (Figures 4D–F). Since RTs in AP trials were slower, we expected to find negative correlations between the RTs and periodicity.

During most of the V time intervals, RTs were consistently ~100 ms slower than the P conditions (Figure 4A) and presented no correlations except during the 0.5 s time-interval (Figure 4D). In other words, regardless to the different aperiodicities, RTs were mostly constant. This is consistent with the perceptual compression phenomenon described in “Vision Compressed the Perceived Duration of Aperiodic Intervals” Section because in spite of the metrics of the stimuli they are perceived of similar duration.

The RTs of the A AP condition slightly increased as a function of time intervals (Figure 4B). Additionally, all of them except the 0.5 s time-interval were negatively correlated to periodicity (Figure 4E). We observed that RTs were more variable as a function of time interval increments, thus, correlations are explained by aperiodicity while the mean RTs are explained by the time interval. In contrast, the P condition showed that RTs decreased during intervals longer than the reference. This result is explained by the observation that the more the subjects spent time perceiving a regular stimulus, the faster they decided their durations.

We compared RTs of the AV P and AV AP conditions to their corresponding V and A distributions to determine what modality contributed the most to the AV results. AV resulted different to V during P; *t*_(185)_ = −2.88, *p* < 0.01, and AP condition; *t*_(185)_ = −6.03, *p* < 0.01. In contrast, AV was different only to A P; *t*_(185)_ = −4.16, *p* < 0.05. Therefore, since AV AP was not different to A AP, these results are explained by an A dominance during discriminations of AP stimuli. Finally, Figure 4F shows that in AV trials, RTs correlations to periodicity index occurred at intervals near threshold, i.e., 0.7 and 0.9 s, and during the supra-threshold time interval of 1.1 s. Given that during those A time intervals correlations also existed, but not during V intervals, then again, RTs of AV AP are explained by an auditory dominance.

### The Reaction Times as a Function of Accuracies Revealed the Visual Long Aperiodic Time Intervals to be Perceptually Compressed

**Figure 5:**
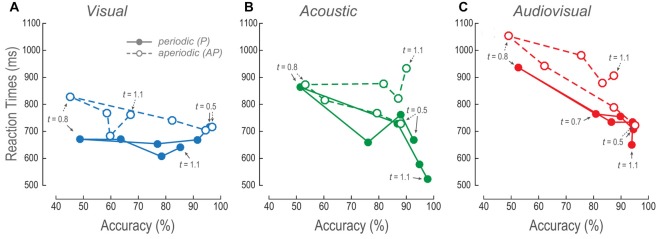
Relationship between RTs and accuracy. **(A–C)** V, A and AV mean RTs as a function of their respective mean accuracies at every time interval. The lines follow the trajectory of P and AP stimuli. Closed circles, continuous lines and arrows: P stimuli. Open circles, dashed lines and arrows: AP stimuli. Arrows signal some of the comparison time intervals.

To further understand the effect of AP stimuli on the overall performance, we plotted the V, A and AV RTs as a function of their respective accuracies (Figure 5). Figure 5A shows the V mean RTs as a function of efficiencies across time intervals. Increments in the P accuracies showed no changes in the RTs. In contrast, RTs of the V AP decreased as a function of accuracy increase for shorter times. However, in time intervals longer than the reference such linear relationship was no longer apparent because accuracies and RTs were almost constant.

The A and AV RTs, for both P and AP conditions, decreased as a function of accuracy increments (Figures 5B,C). Nevertheless, AP conditions presented higher RTs during time intervals longer than the reference. These results were concordant with the previously described results where the longer the time intervals, the longer the RTs.

Altogether, these observations suggest that both measures of performance (i.e., accuracy and RTs) proved that V AP stimuli were considered to last shorter as compared to the same V P time intervals. Furthermore, V behavioral dynamics were consistent to a perceived compression of time intervals as compared to the A AP, and AV AP conditions.

## Discussion

We performed a V, A and AV time-interval discrimination of AP patterns to test whether the irregularity of such patterns affected their perceived durations. Our results prove that, indeed, AP stimuli affected estimated durations. V interval perception was compressed, i.e., accuracies worsened during intervals longer than the stimulus of reference. Meanwhile, A and AV accuracies decreased globally for both, lower and higher than the reference time intervals. Moreover, AV estimations were explained by auditory dominance rather than by multisensory integration.

### The Visual Estimates of Time Intervals Are Compressed by Aperiodic Stimuli

In psychophysical performances within our tests, we observed that AP intervals produced perceptual time compressions because subjects frequently reported comparisons to be shorter than the reference. However, someone could argue that subjects discriminated AP stimuli based on a perceived reduction of the number of pulses (i.e., <6 pulses) rather than on the duration of the patterns. That could have been the case if, at times, aperiodicity had arranged some pulses together to create a flicker fusion phenomenon. The fusion phenomenon was previously observed during presentations of looming discs surrounded by static discs (van Wassenhove et al., 2008). Nevertheless, in those experiments, visual stimuli were perceived as dilated. Hence, fusing pulses would increase perceptual durations as in studies where large V stimuli were perceived as longer (Xuan et al., 2007). Another possibility could have been that consecutive pulses created an increase in the perceptual frequency. Still, dilation phenomena were previously reported (Kanai et al., 2006). In our study, we intended to avoid those possibilities by restricting the appearance of two consecutive pulses above V-fusion frequency at 15 Hz. Nevertheless, if regardless of our restrictions, V-fusion occurred, then pulse-counting and interval-estimating would have been hard to discern. Furthermore, periodicity and performance would have correlated at every time interval. However, no V correlations arose during intervals shorter than the reference, even when at those time intervals perceptual time compression did happen. Additionally, when V comparisons and reference lasted the same, the performance did not correlate to periodicity, but again, a perceptual time compression was still observed. Lastly, when we evaluated the RTs as a function of the accuracies, we found that V P differed to the A and AV conditions so that RTs did not decrease as a function of the efficiencies. Nevertheless, it is still not plausible to interpret such result as a fusion phenomenon, since V P accuracies were not different to the A and AV accuracies. Consequently, we lean towards disregarding V-fusion or a perceptual increase of frequencies as explanations for the compressed V estimations.

Moreover, given that neither the V AP RTs nor the accuracies were chaotic, e.g., their standard deviations were narrower as compared to the A and AV conditions, suggests that the subjects arrived to time interval estimations faster than during the A AP and AV AP conditions. This result, together to the fact that during V AP condition, time intervals longer than the reference presented invariant and lower RTs and accuracies as compared of those of the A AP and AV AP, is consonant to other studies that proved perceived compressions of V time intervals (Wearden et al., 1998; van Wassenhove et al., 2008; Grondin and McAuley, 2009) but different to others where the increase of the complexity of the V stimuli led to an increase in the interval perception (Schiffman and Bobko, 1974).

### Acoustic Time Interval Estimates Are Affected by Aperiodic Stimuli

Consistent with the principle of sensory appropriateness, audition is better than vision when it comes to judging time intervals (Welch and Warren, 1980). In our experiment, audition was no exception to this rule, proving to be more apt in estimating P and AP time intervals as compared to V. The reason is that A follows a broad range of temporal modulations (Bendor and Wang, 2007; Lemus et al., 2009a,b). However, high instant frequencies can also produce perceptual speed up of events that consequently are perceived as shorter (Wearden et al., 1998). Along with these previous considerations, interval estimations correlated to aperiodicity when the comparisons lasted longer than the reference (except at 1.1 s). This finding is similar to what has been observed in other studies where the increase in the regularity of auditory signals lead to more accurate temporal estimations (Povel and Essens, 1985; Hébert and Cuddy, 2002; Grube and Griffiths, 2009). However, no correlations presented when comparisons were shorter than the reference. The reason is that short intervals do not present a great AP variability, therefore correlations did not occur. Yet, during short intervals, a worsened performance was observed and this slightly affected the overall performance. Such disruptions on interval estimations are consistent with previous results (Goldstone and Goldfarb, 1964; Sakai et al., 1999; Pashler, 2001; Grondin and McAuley, 2009; Rammsayer, 2014; however, unlike the present study, in those experiments aperiodicity was not quantified. As for the A RTs, the fact that during P condition intervals longer than the reference decreased is consistent with previous findings where subjects spending more time perceiving a regular stimulus resulted on more accurate discriminations (Luna et al., 2005).

### Audiovisual Time Interval Estimation Is Dominated by Audition

Aperiodicity also affected AV conditions where differences with the P control occurred at the threshold to the reference stimuli. The AV AP slope was different from the AV P slope. Such an effect is clearly due to threshold AP discriminations.

In agreement with other studies, most effects observed during AV conditions are consistent with audition dominance (Repp and Penel, 2002; Grahn et al., 2011). Nonetheless, during 1.0 s and 1.1 s comparisons, performance correlated slightly better to periodicity, as compared to A, but still, accuracy was not different. It could be argued in this case, that V created a bias, such as the one reported by van Wassenhove et al. (2008). However, at intervals of 1 and 1.1 s, AV accuracy was similar to A (but slightly more variable), and not to V. Thus, we conclude that no multisensory integration occurred because performance did not improve.

The RTs analyses showed AV to be different to V for the P and AP conditions. However, AV was different to A only during the P condition. A plausible interpretation could be that the subjects did not perceive the superimposed AV stimuli as a unit, as in other results where desynchronized V and A cues impaired V timing (Kösem et al., 2012). Nonetheless, the subjects did not report any discrepancies because A stimuli shifted only 90 ms as related to V, and therefore, we have no reason to suspect any perceptual asynchrony (Dixon and Spitz, 1980). It would be interesting to further investigate how non-periodic stimuli would affect AV perceptual synchrony. Furthermore, cross-modal experiments could also address this question. Thus, instead of superimposing A and V stimuli, the reference and comparison stimuli should be of different modalities, benefitting from the best of each modality when estimating time intervals.

Our results show differences between A and V estimations of the time intervals of non-periodic stimuli, that are consistent with models of independent sensory processing (Hébert and Cuddy, 2002; Karmarkar and Buonomano, 2007). In those models, non-linear time metrics occur for short time intervals, like those used in the present study. Even when our results are difficult to adjust into neuronal models, they are consistent with other studies where AP stimuli are coded by different cortical areas (Luna et al., 2005; Rossi-Pool et al., 2016), and to studies in interval synchronization-continuation tasks that show that the firing rate of neurons in the supplementary motor cortex accumulate information from regular, but not from AP stimuli (Bengtsson et al., 2009; Merchant et al., 2011). In the context of our study, this could mean that the sampling rate of the auditory modality is capable of tracking AP time intervals, whereas the V system gets saturated, thus resulting in time interval estimations to be compressed.

The aim of the present study was to establish whether AP patterns of V, A and AV stimuli were perceived to last differently than the controls of regular structures. We used a simple method for quantifying the structure of the dynamics of AP patterns and compared them to the perceived duration of the stimuli. Our results show that aperiodicity produces a compression of the time interval estimations of V, but not A nor AV stimuli. Interestingly, no behavioral advantage was observed during AV discriminations, which suggests that time interval perception is created within each sensory pathway and not in association cortical areas. Future neurophysiological experiments in behaving animals may test such hypothesis.

## Author Contributions

FD and LL designed the experiments. FD performed the experiments and analyzed the data. LL contributed to data analysis. FD and LL wrote the article.

## Conflict of Interest Statement

The authors declare that the research was conducted in the absence of any commercial or financial relationships that could be construed as a potential conflict of interest.

## Abbreviations

A: acoustic
AP: aperiodic
AV: audiovisual
P: periodic
RT: reaction time
V: visual.
